# Tickborne *Neoehrlichia mikurensis* in the Blood of Blood Donors, Norway, 2023

**DOI:** 10.3201/eid3111.250125

**Published:** 2025-11

**Authors:** Hanne Quarsten, Charlotte N.B. Ryen, Linn K.T. Mørk, Christine Wennerås, Christine T. Steinsvåg

**Affiliations:** Sørlandet Hospital, Kristiansand, Norway (H. Quarsten, C.N.B. Ryen, L.K.T. Mørk, C.T. Steinsvåg); Sahlgrenska Academy at the University of Gothenburg and Sahlgrenska University Hospital, Göteborg, Sweden (C. Wennerås)

**Keywords:** Neoehrlichia mikurensis, bacteria, tick-borne infection, vector-borne infections, blood donors, transfusion-transmitted infection, immunosuppression, Norway

## Abstract

The tickborne bacterial pathogen *Neoehrlichia mikurensis* has been detected in <1% of blood donors in Sweden. *N. mikurensis* can give rise to asymptomatic persistent infections. Up to 25% of *Ixodes ricinus* ticks in southern Norway are infected with *N. mikurensis*. We investigated the incidence of *N. mikurensis* infection among blood donors in this region. We detected *N. mikurensis* in the blood of 45/499 (9%) blood donors by independent PCR methods; 69% of those were repeatedly positive 1–7 months after the first detection and tested negative after doxycycline treatment. We tested 8/19 adult recipients of potentially infected blood; none tested positive for *N. mikurensis* at the time of testing (191–301 days after transfusion). Our study identified a very high rate of infection with *N. mikurensis* in blood donors in Norway; whether infection can be transmitted by transfusion of blood products, however, remains unclear.

*Neoehrlichia mikurensis* is the cause of the infectious disease neoehrlichiosis. This tickborne bacterium is widespread in Europe and northern Asia. *Ixodes ricinus* ticks are the main vector in Europe. Infection prevalence varies from barely detectable to >20% (*1*,*2*); the southern coastline of Norway is a high-prevalence region, where up to 25% of ticks are infected with *N. mikurensis* (*3*).

*N. mikurensis* can cause a long-lasting infection with spiking fever. Immunosuppressed patients, predominantly those who have been treated with rituximab (anti–CD20/B cell) or splenectomized, are at risk of developing severe neoehrlichiosis (*4*). The target of this intracellular species of bacteria is human vascular endothelium (*5*). Severe neoehrlichiosis may be accompanied by vascular events such as venous thromboembolism in immunocompromised persons and arteritis in immunocompetent persons (*6*). Molecular detection of *N. mikurensis* DNA by PCR is the only diagnostic test available because antibody tests have not yet been established and *N. mikurensis* does not grow in routine blood culture; it might grow in specialized cell culture. Neoehrlichiosis is underrecognized because of low awareness among clinicians and limited availability of microbiologic diagnostic methods (*4*,*7*).

A growing body of evidence suggests that *N. mikurensis* may give rise to asymptomatic infections, presumably persisting for months (*8*–*11*). It is not clear if persons with persistent asymptomatic *N. mikurensis* infection are at risk for activation of acute disease at a later stage. Carriage of *N. mikurensis* in the blood of asymptomatic healthy persons raises the possibility of *N. mikurensis* transmission through blood transfusion. Two retrospective studies have examined that issue. A study of blood donors in Sweden found that 0.7% of the donors had detectable levels of *N. mikurensis* DNA in the blood, yet no transmission of the infection could be established (*12*). A study of blood donors in Denmark found that none were infected by *N. mikurensis* (*13*).

The aim of this study was to investigate the prevalence of *N. mikurensis* in healthy blood donors living in southern Norway, an area highly endemic for *N. mikurensis* infection, and to analyze recipients of blood components from infected donors for possible infection with *N. mikurensis*. The Norwegian Regional Committee for Medical and Health Research Ethics, South-Eastern region, approved the study (reference no. 513442). All blood donors and blood recipients provided written informed consent, which included permission to collect information by approved questionnaires and to collect blood and analyze it for tickborne infections.

## Materials and Methods

### Blood Donors and Blood Components

We recruited blood donors at the Department of Immunology and Transfusion Medicine at Sørlandet Hospital (Kristiansand, Norway) during March 20–June 15 (group 1, n = 381) and August 21–September 14, 2023 (group 2, n = 118) when they attended the hospital to donate blood. Each week, we invited all donors attending Monday–Thursday until up to 40 donors (usually ≈10 per day) were recruited per week. Blood donors answered a questionnaire at the time of study inclusion regarding health complaints the preceding year—pain, headache, dizziness, fever, night sweats, sleep problems, nausea or digestive problems, fatigue, and rash—as well as history of tick bites, tickborne infections, and antimicrobial treatment.

We collected EDTA blood and serum from all participants. Blood components (red blood cell or platelet concentrates) were produced in accordance with standard procedures. All blood components were leukocyte-reduced. No pathogen reduction technology was used.

### DNA Extraction

We isolated DNA by MagNAPure 96 DNA or Viral NA Small Volume Kit (Roche, https://www.roche.com) from 200 µL of whole blood and plasma/buffy coat fractions of the blood from patients 1–4 days after sampling and plasma/buffy coat fractions of the blood from blood donors within 1 day of sampling. We collected plasma and buffy coat after centrifugation at 1,000 × *g* for 12 minutes and concentrated to 200 µL by centrifugation at 10,000 × *g* for 2 minutes. We added MS2 bacteriophage DNA to all samples before extraction to monitor for integrity of extraction and amplification by MS2-specific PCR (*14*). If the internal control system indicated inefficient DNA isolation from the plasma/buffy coat fractions of the blood donors, we performed additional extractions from thawed whole blood. We analyzed all index sample DNA after storage at −20°C; we tested some of the other samples before freezing.

### Real-Time PCR

We tested all samples by real-time PCR for *N. mikurensis*; we conducted 2 independent assays targeting the *groEL* gene. The sequences, concentrations, and thermocycling conditions of the assays have previously been published (*8*). The protocols used 5 µL of DNA in a 15-µL reaction mixture consisting of 5 mM MgCl_2,_ 0.5 units uracil DNA-glycosylase (Eurogentec S.A., https://www.eurogentec.com), and LightCycler FastStart DNA master mix (Roche) with primers and probes. The PCRs are validated for human diagnostics and are in routine use, 1 (CNM-I) at the national reference laboratory for *N. mikurensis* diagnostics at Sahlgrenska University Hospital (Göteborg, Sweden) and 1 (CNM-II) at the national reference laboratory for *Borrelia* diagnostics at Sørlandet Hospital, the site of this study. The CNM-I assay had been validated by using specimens from ≈180 patients in Sweden with confirmed neoehrlichiosis and 180 negative controls; all samples were verified by panbacterial 16S rRNA PCR and sequencing. The CNM-I and CNM-II assays show complete concordance with neoehrlichiosis patients in Norway, as demonstrated in a previous study involving 12 persons diagnosed with neoehrlichiosis (*8*).

We analyzed blood donor samples 1 time by each real-time PCR. If only 1 of the PCR methods was positive, we retested samples in triplicate by both methods. When >1 replicate was positive in the triplicate testing, we considered the donor positive. We tested confirmatory and posttreatment samples of blood donors and samples from blood recipients by both methods in triplicate.

### Follow-Up of *N. mikurensis*–Positive Blood Donors

We informed blood donors by letter if *N. mikurensis* was detected in their blood and asked them to provide a new sample for retesting. If we detected *N. mikurensis* in a repeat blood sample, we treated the donors with antimicrobial drugs, retested them by PCR after completion of the course of treatment to ensure they had cleared the infection, and asked them to answer the enrollment questionnaire at the time of follow-up and posttreatment sampling.

### Blood Recipients

All adult recipients of red blood cell or platelet components from *N. mikurensis*–positive donors who were alive at the time of the study received written information and invitation to participate in the study. We did not contact plasma recipients because only batch-processed virus-inactivated plasma (Octaplasma; Octapharma, https://www.octapharma.com) is used for transfusion in Norway, not single-donor plasma. We offered all blood recipients PCR testing, either for their participation in the study or as part of routine diagnostic procedure. Children <18 years of age were not eligible to participate in the study; however, we informed the attending hospital pediatrician if a child had been transfused with a blood component from a *N. mikurensis*–positive donor. We collected EDTA blood and serum from the participating blood recipients. The included patients answered a questionnaire regarding health complaints the preceding year, immune status, medication, cardiovascular events, and history of tick bites, tickborne infections, and antimicrobial treatment.

### Statistics

We used χ^2^ (Table 1) and Fisher exact (Table 2) tests to compare categorical variables. We used statistics calculators (Social Science Statistics, https://www.socscistatistics.com) to perform Mann-Whitney U test to compare age and sex in different groups and analysis of variance for pairwise comparison to examine >2 means. We considered p values <0.05 to be significant.

**Table 1:** Seasonal distribution of *Neoehrlichia mikurensis* DNA found in blood donors, Norway, 2023*

Sample	No. (%) *Neoehrlichia mikurensis* DNA–positive blood donors			
	March–April, n = 175	May–June, n = 206	August–September, n = 118	p value
Index sample	17 (9.7)	16 (7.8)	12 (10)	0.71
Follow-up sample	12 (6.9)	12 (5.8)	7 (5.9)	0.91
Difference, index to follow-up	5 (2.9)	4 (1.9)	5 (4.2)	0.63

*p values determined by χ^2^ test.

**Table 2:** Characteristics of blood donors at study inclusion and after treatment with antimicrobial drugs in study of tickborne *Neoehrlichia mikurensis* in the blood of blood donors, Norway, 2023*

Reported data	No. (%) blood donors		p value
	*N. mikurensis* DNA negative	*N. mikurensis* DNA positive	
Tick bite in current year	97/454 (21)	16/45 (36)	0.04
History of tick bites	370/454 (82)	40/45 (89)	0.31
History of borreliosis/TBE†	38/454 (8.4)	6/45 (13)	0.27
Health complaints in previous 4 months			
Pain	10/451 (2.2)	1/44 (2.3)	>0.99
Headache	6/451 (1.3)	2/44 (4.6)	0.15
Dizziness	3/451 (0.7)	0/44 (0)	>0.99
Fever	0/451 (0)	0/44 (0)	>0.99
Night sweat	8/451 (1.8)	2/44 (4.6)	0.22
Sleep problems	11/450 (2.4)	1/44 (2.3)	>0.99
Nausea/digestive problems	0/451 (0)	0/44 (0)	>0.99
Fatigue	22/451 (4.9)	3/44 (6.8)	0.48
Rash	4/450 (0.9)	0/44 (0)	>0.99
Improvement of health complaints after treatment with antimicrobial drugs			
Pain		3/29 (10)	
Headache		4/29 (14)	
Dizziness		2/29 (6.9)	
Fever		1/29 (3.5)	
Night sweat		2/29 (6.9)	
Sleep problems		4/29 (14)	
Nausea/digestive problems		2/29 (6.9)	
Fatigue		3/29 (10)	
Rash		1/29 (3.5)	

*p values determined by Fisher exact test. TBE, tickborne encephalitis. †History of borreliosis/TBE; 25 were reported as borreliosis, 19 were unspecified (either borrelioses or TBE) and none reported TBE.

## Results

### Blood Donors

The study included 499 blood donors, most of them living near or in Kristiansand city on the southern coast of Norway. The male-to-female ratio was 1.03:1, and the median age was 44 years (range 18–66 years). Most (82%) of the blood donors had a history of tick exposure. We noted no age-related difference in self-reported tick exposure among the blood donors; the average age was 42 years in those who reported no tick bites (n = 89), 41 years in those who reported 1–2 tick bites (n = 144), and 43 years in those who reported >2 tick bites (n = 266) over their lives. We observed no significant difference between the groups (p = 0.21).

### Detection of *N. mikurensis* in Blood Donors

We analyzed DNA from the plasma/buffy coat (n = 490) or whole blood (n = 9) from blood donors by 2 different real-time PCR methods for detection of *N. mikurensis*. We detected *N. mikurensis* DNA in 45/499 (9.0%) of the blood samples (Figure). All 45 blood donors had PCR cycle threshold values >34. We first tested blood donor group 1 (n = 381) for *N. mikurensis* infection by PCR 2–5 months after the blood samples were drawn and group 2 (n = 118) 0–1 month after the blood samples were drawn (Table 1). The rate of *N. mikurensis* detection in index samples was the same in blood donors in early spring (March–April, 12/17 [71%]) as in late summer/autumn (August–September, 7/12 [58%]) (Table 1). In follow-up samples, drawn 1–7 months after the index sample, *N. mikurensis* DNA was detectable in 31/45 persons. The rate of donors repeatedly positive in follow-up sampling did not differ significantly in different seasonal periods; however, it was slightly lower among donors recruited in late summer (August–September). Out of the 31 repeatedly positive blood donors, 14 (45%) reported receiving tick bites between the first and second blood sampling, most during June–September.

**Figure:**
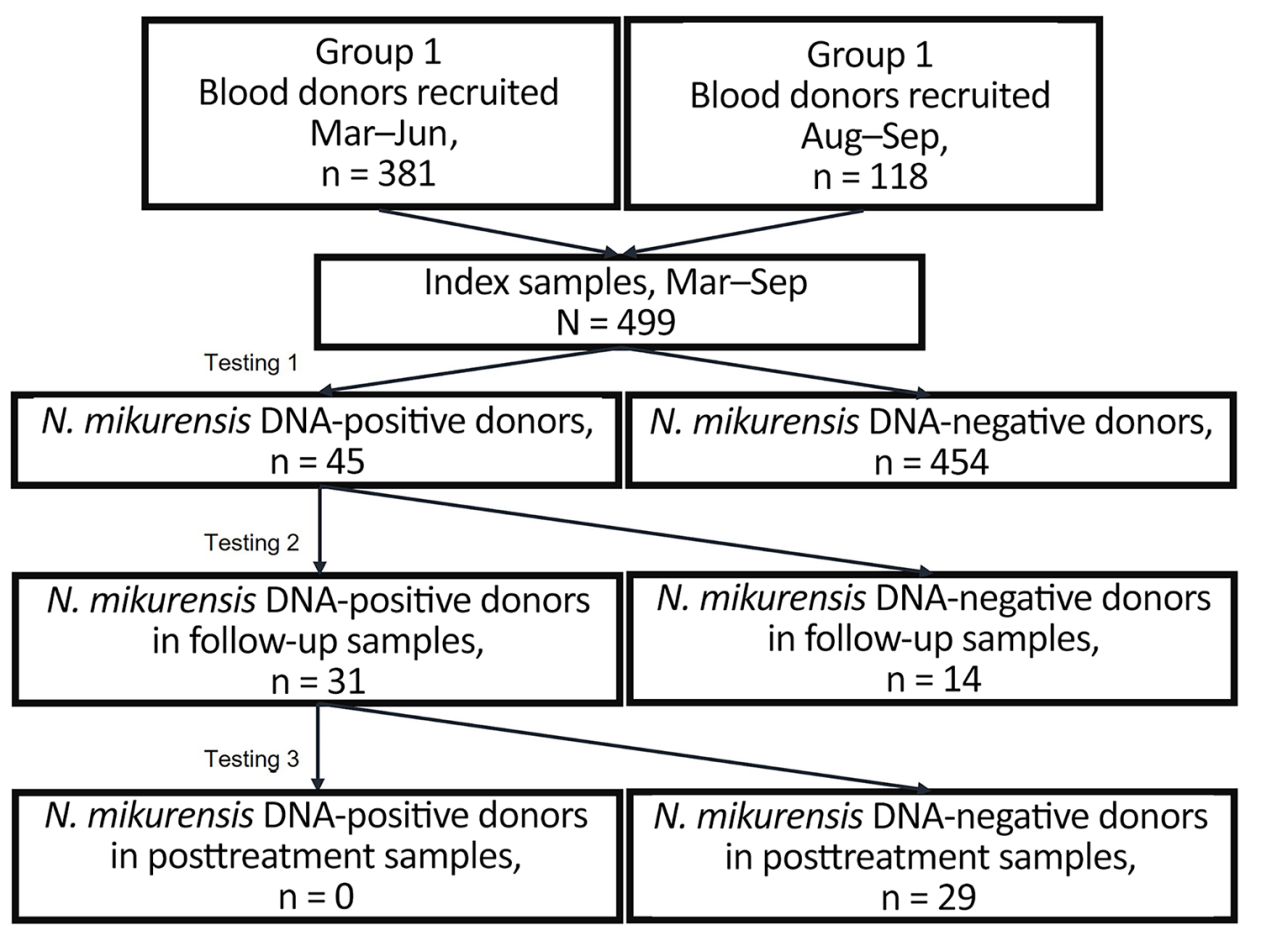
Flowchart of the study design and follow-up testing of *N. mikurensis–*infected blood donors in study of tickborne *Neoehrlichia mikurensis* in the blood of blood donors, Norway, 2023. Two patients did not provide blood samples for posttreatment testing.

### Characteristics of Negative and Positive Blood Donors

The blood donors who tested positive for *N. mikurensis* were older (median age 52 years) than the blood donors who tested negative (median age 42 years) (p<0.001). We observed no significant difference by sex; 18/246 female and 27/253 male donors tested positive (p = 0.19). Moreover, the *N. mikurensis* positive group reported more tick bites in the year of the study than the noninfected group (p = 0.04) (Table 2). However, we observed no significant difference in overall history of tick exposure, history of Lyme borreliosis or tickborne encephalitis, or experienced health complaints between the groups (Table 2).

### Follow-up of *N. mikurensis*–Positive Blood Donors

None of the infected donors experienced fever at the time of first blood sampling and donation. We offered treatment with doxycycline (200 mg/d for 3 weeks) and retesting by PCR after treatment to the 31 blood donors with *N. mikurensis* DNA detected in 2 repeated blood samples. We retested most (29/31) blood donors and did not detect *N. mikurensis* DNA in any of them. Some reported improvement in a few health complaints, but more than half (16/29) experienced no change in health after treatment (Table 2).

### Testing of Recipients of Blood Components from *N. mikurensis* Positive Donors

We traced recipients of blood components donated by *N. mikurensis* PCR-positive donors. Two platelet concentrates from 1 of the donors had been sent to another hospital, but it is unknown whether they were transfused. Thirty-two of the 45 positive donors provided 39 blood component units that were given to 37 unique patients. Thirteen recipients had died by the time we traced the donations; we did not explore their cases further. Of the remaining 24 blood recipients, 5 were children and could not be included in the study. All of the remaining 19 patients had received red blood cell concentrates; 17 of them had received 1 unit, 1 of them 2 units from the same donor, and 1 recipient 1 red blood cell and 1 platelet concentrate from 2 different donors. The median time from donation to transfusion was 20 days (range 5–34 days). We invited all 19 recipients to participate in the study; 8 recipients provided blood samples for testing. Each had received 1 red blood cell concentrate from an infected donor. One had also received 1 platelet unit on a different date. Median time from transfusion to testing of recipients was 246 days (range 196–301 days). We did not detect *N. mikurensis* in any of the recipients. The median age of the 8 transfused patients was 69 years (range 27–79 years). None were immunosuppressed or reported fever at the time of blood sampling. Two patients experienced venous thrombosis, 1 patient in the arm about the same time as receiving blood and 1 few months after transfusion, but both tested negative for *N. mikurensis*. Five recipients self-reported treatment with antimicrobial drugs; 1 received treatment on 2 occasions. Three patients were treated with dicloxacillin, apocillin, and clindamycin, which are not effective against *N. mikurensis*. The type of drugs was not specified at 3 other occasions for 3 different patients. We could not exclude treatment with drugs active against *N. mikurensis*.

## Discussion

We found high incidence (9%) of infection with *N. mikurensis* among blood donors from the Kristiansand region in southern Norway. We treated all repeatedly PCR positive blood donors (69%) with doxycycline; all tested negative after treatment. We did not establish transmission of infection in any of the recipients of blood components from the infected donors who were available for testing.

The observed rate of *N. mikurensis* infection was considerably higher than that reported in previous studies of blood donors in Sweden (0.7%) (*12*) and Denmark (0%) (*13*). The high rate of *N. mikurensis* in blood donors in our study may be partly explained by our region being highly endemic for *N. mikurensis* infections and with <25% *N. mikurensis*–infected ticks (*3*). The prevalence of *N. mikurensis* in ticks in the other Scandinavian countries varies (0% to >10%) and differs across locations (*5*,*15*,*16*). The donors in Sweden were recruited from 1 region (Kalmar); the blood donors in Denmark were recruited from all over Denmark. Most areas in Denmark have low prevalence, 0%–5%, of *N. mikurensis* infection in ticks (*15*).

More than 80% of the blood donors in our area had a history of tick bite, similar to the cohort of blood donors in Sweden (*12*). The high *B. burgdorferi* sensu lato seroprevalence of 18% in the blood donor population and 22% in the adult population underscore the high exposure to ticks in the Kristiansand region (*17*,*18*). The prevalence of *N. mikurensis* antibodies in the population is, however, still unknown because of the lack of serologic assays for this emerging pathogen.

The very low (0.7%) or nonexistent prevalence of *N. mikurensis* in previous studies of blood donors (*12*,*13*) may reflect methodological issues regarding the type of blood tested (serum, plasma, or whole blood). The high prevalence of *N. mikurensis* infection in our study is to a certain extent a result of our strategy of isolating DNA from the buffy coat fraction of fresh whole blood. In that fraction, bacteria and leukocytes are concentrated, and the diagnostic sensitivity of *N. mikurensis* detection is optimized (*19*). Testing of plasma, serum, and frozen or nonconcentrated whole blood material is suboptimal and may reduce the overall sensitivity of the testing; those factors may affect the results of other studies on blood donors.

More than two thirds (31/45) of initial positive blood donors from Kristiansand were repeatedly positive for *N. mikurensis* DNA for up to 7 months after the first detection. This finding strengthens the notion that *N. mikurensis* may be associated with an asymptomatic carriage state, as reported in other studies (*8*,*10*–*12*). The infection rate among blood donors was as high in March–April, the first spring months in Norway when it is still cold and the vegetation is low, as in August–September when the grass is high and tick bites are more difficult to avoid. That finding indicates that a substantial proportion of donors had an ongoing infection from a previous tick bite rather than a recent one. Alternatively, a second positive test in donors may in a few cases be explained by reinfection; nearly half of the repeatedly positive donors reported a new tick bite between the index and follow-up samples. The tendency of *N. mikurensis* to cause a persistent infection will lead to an accumulation of positive persons over time, which may result in the high infection rates seen among blood donors and immunosuppressed patients in the Kristiansand region (*8*).

The *N. mikurensis*–infected blood donors did not report any signs of infection and were allowed to donate blood when they attended the blood bank. According to national guidelines in Norway, donors reporting an asymptomatic tick bite are prevented from donating blood for 4 weeks, whereas donors who have been treated for Lyme borreliosis must refrain from donation for 6 months. Our study shows that the current guidelines will not prevent *N. mikurensis*–infected blood donors from donating blood. Blood donors reported some common health complaints at the time of donation regardless of whether they tested positive for *N. mikurensis*. Headache and nightly sweats were more frequent in the infected donors but did not reach statistical significance. The donors who repeatedly tested positive for *N. mikurensis* DNA did not donate blood again until the *N. mikurensis* infection was eradicated by antimicrobial drug treatment. Some of the treated donors experienced relief from various types of reported health complaints after antimicrobial treatment. We could not verify in this study whether those results were causally related to *N. mikurensis* infection. Furthermore, antimicrobial therapy may have beneficial immunomodulatory effects besides killing of bacterial pathogens (*20*).

In our study, none of the recipients of blood from the infected blood donors tested positive for *N. mikurensis* by PCR. However, we tested only 8 of 19 traced recipients. Furthermore, we did not test the blood components of the *N.* mikurensis–infected blood donors for *N. mikurensis*. Our findings are consistent with the Sweden study in which 7 recipients of potentially *N. mikurensis*–infected blood also tested negative (*12*). The time from transfusion to testing of the recipients in both our study and the Sweden study was 2–13 months. The delayed testing might have reduced the probability of detecting transmission of infection because of either spontaneous recovery or adequate antimicrobial treatment. Acute disease caused by *N. mikurensis* has not been reported in children (*21*); we did not test the children receiving blood from *N. mikurensis* infected donors in this study.

All recipients of blood in our study received transfusions of leukocyte-reduced components. Leukocyte reduction of cellular blood components has been shown to lower the risk for transmission of tickborne *Anaplasma phagocytophilum*, which is known to infect granulocytes, although experimental models indicate that the risk is not eliminated (*22*,*23*). *N. mikurensis* is an intracellular bacterium with tropism for endothelial cells, similar to most *Rickettsia* spp. (*5*,*24*). In blood components, *N. mikurensis* might be present inside circulating endothelial cells, phagocytosed by neutrophils, or free in blood (*5*,*25*). In general, bacteria contaminating blood components may be removed by leukocyte reduction if they are phagocytosed or inside of cell types that aggregate in the leukocyte fraction or, if filtration is used in the process, if they attach to the filter matrix (*26*).

Red blood cell components are stored at 4°C. At low temperatures, contaminating extracellular bacterial species are not likely to replicate and their survival rate is often reduced. However, the intracellular bacterial pathogen *R. conorii* not only survived for 35 days in canine blood components stored at 4°C but also breached the leukocyte filter used and maintained infectivity (*27*). Platelet components are stored at room temperature, which increases the risk for bacterial growth and transmission of infection. Survival of *N. mikurensis* in blood components should be investigated to clarify the ability of *N. mikurensis* to be transmitted via blood transfusion.

Immunosuppression, in particular B cell–depleting therapy (rituximab) or splenectomy, is the main risk factor for developing acute neoehrlichiosis (*21*). None of the study recipients traced after receiving blood products from *N. mikurensis*–infected donors were immunosuppressed and at a substantial risk of contracting disease. This condition could partly explain why *N. mikurensis* was not detected in their blood after transfusion. Still, we note that immunocompetent persons may also be vulnerable and in danger of contracting severe infection, given that 20% of neoehrlichiosis patients in Sweden were not immunocompromised (*6*,*21*).

It is too early to decide whether *N. mikurensis* can be transmitted by blood transfusion. However, the high incidence of *N. mikurensis* among blood donors in our highly endemic area is an alarming finding that requires further study. Many factors could influence the risk for transmission and the putative establishment of persistent infection with *N. mikurensis* bacteremia among blood recipients; among those are the ability of this emerging bacterial pathogen to stay alive during preparation and storage of the blood components.
